# Comparative muscle transcriptome associated with carcass traits of Nellore cattle

**DOI:** 10.1186/s12864-017-3897-x

**Published:** 2017-07-03

**Authors:** Bárbara Silva-Vignato, Luiz L. Coutinho, Aline S. M. Cesar, Mirele D. Poleti, Luciana C. A. Regitano, Júlio C. C. Balieiro

**Affiliations:** 1 College of Animal Science and Food Engineering, University of São Paulo, Pirassununga, SP 13635-900 Brazil; 2 College of Agriculture “Luiz de Queiroz”, University of São Paulo, Piracicaba, SP 13418-900 Brazil; 3 Embrapa Pecuária Sudeste, São Carlos, SP 13560-970 Brazil; 4 College of Veterinary Medicine and Animal Science, University of São Paulo, Pirassununga, SP 13635-900 Brazil

**Keywords:** Backfat thickness, *Bos taurus indicus*, Ribeye area, RNA-Seq

## Abstract

**Background:**

Commercial cuts yield is an important trait for beef production, which affects the final value of the products, but its direct determination is a challenging procedure to be implemented in practice. The measurement of ribeye area (REA) and backfat thickness (BFT) can be used as indirect measures of meat yield. REA and BFT are important traits studied in beef cattle due to their strong implication in technological (carcass yield) and nutritional characteristics of meat products, like the degree of muscularity and total body fat. Thus, the aim of this work was to study the *Longissimus dorsi* muscle transcriptome of Nellore cattle, associated with REA and BFT, to find differentially expressed (DE) genes, metabolic pathways, and biological processes that may regulate these traits.

**Results:**

By comparing the gene expression level between groups with extreme genomic estimated breeding values (GEBV), 101 DE genes for REA and 18 for BFT (false discovery rate, FDR 10%) were identified. Functional enrichment analysis for REA identified two KEGG pathways, MAPK (Mitogen-Activated Protein Kinase) signaling pathway and endocytosis pathway, and three biological processes, response to endoplasmic reticulum stress, cellular protein modification process, and macromolecule modification. The MAPK pathway is responsible for fundamental cellular processes, such as growth, differentiation, and hypertrophy. For BFT, 18 biological processes were found to be altered and grouped into 8 clusters of semantically similar terms. The DE genes identified in the biological processes for BFT were *ACHE, SRD5A1, RSAD2* and *RSPO3*. *RSAD2* has been previously shown to be associated with lipid droplet content and lipid biosynthesis.

**Conclusion:**

In this study, we identified genes, metabolic pathways, and biological processes, involved in differentiation, proliferation, protein turnover, hypertrophy, as well as adipogenesis and lipid biosynthesis related to REA and BFT. These results enlighten some of the molecular processes involved in muscle and fat deposition, which are economically important carcass traits for beef production.

**Electronic supplementary material:**

The online version of this article (doi:10.1186/s12864-017-3897-x) contains supplementary material, which is available to authorized users.

## Background

Meat is the most important source of animal protein for the human diet; it consists mainly of skeletal muscle, and of varying amounts of connective tissue, implicated on its qualitative and quantitative characteristics, as well as small amounts of epithelial and nervous tissues. Meat represents the edible portion of the carcass, in other words, the part that will be destined for the final consumers and can be represented by the yield of commercial cuts [1, 2].

Commercial cuts yield is economically important since it affects the final value of the products due to the proportion of fat, muscle, and bone in the carcasses. The direct determination of meat yield is difficult in practice, therefore the measures of ribeye area (REA) and backfat thickness (BFT), sections of the *Longissimus dorsi* muscle, are often used as indirect measures of this trait [3–5].

REA and BFT are well studied traits in beef cattle due to their implication in technological and nutritional characteristics of meat products. The ribeye area is used as an indicator of degree of muscularity, edible mass of carcass and yield of cuts with high commercial value. This measure can also be associated with the length and weight of the carcass (hot carcass weight) [3, 6, 7].

The amount of BFT deposited on the carcass is related to the total body fat and plays a major role in beef’s flavor and juiciness, which is directly associated with production costs. In the meat industry, an adequate layer of fat acts as a thermal insulator during carcass cooling process, avoiding problems such as cold shortening [8, 9]. Also, the layer of fat is an important source of essential fatty acids and acts in the transport of fat-soluble vitamins, constituting a source of energy and insulation for the body of the animal [10].

Selection based on body composition, particularly on the relative proportion of muscle and fat in the carcass, is critical in meat-producing animals [5, 11]. Most carcass traits have moderate to high heritability, indicating that the selection may result in significant genetic progress [5]. According to Costa et al. [12] and Clímaco et al. [10], feedlot finished zebu breeds may present the same proportion of edible portion as other genotypes (crosses with taurine breeds), and even greater muscularity and higher carcass yield.

Several tools have been developed to improve the accuracy of animal selection and thus improve economically important traits in beef cattle, such as large-scale genotyping platforms, high-density panels of single nucleotide polymorphisms (SNP), and genome-wide association studies (GWAS). Besides these, many studies have used RNA Sequencing (RNA-Seq) to unravel complex traits in production animals. This high-throughput technology has been successfully employed in beef cattle for traits such as muscle development, intramuscular fat, and fatty acid profile, with interesting results of the phenotypic differences within and between populations [13–20].

Meat quality and carcass traits are influenced by a complex network of gene interactions in the muscle [21]. Therefore, elucidating the relationships between genes and how these genes, in turn, influence the carcass traits is critical for understanding the development of the animals, as well as the biological processes (BP) and metabolic pathways that may influence the final amount of fat and muscle in the carcasses.

Tizioto et al. [22] working with this same population of Nellore steers, identified six QTL (quantitative trait loci) that individually explained 0.8% of the additive genetic variance of REA, and a QTL that explained 0.36% of the variation in BFT. Gomes et al. [23] reported that SNPs in genes related to protein turnover, like genes regulating the ubiquitin-proteasome system, may be associated with growth and carcass traits in bovine. Junior et al. [24] found SNP-windows located on chromosomes 5, 6, 7, 8, 10, 12, 13, 16, 17, 20, 24 and 29 that together explained 8.72% and 11.38% of the genetic variance for REA and BFT in a population of Nellore cattle.

Despite those studies, there are still gaps to be filled about the molecular mechanisms that regulate carcass traits in cattle. Thus, the aim of this work was to study the *Longissimus dorsi* muscle transcriptome of Nellore cattle, associated with ribeye area and backfat thickness, to find differentially expressed genes, metabolic pathways, and biological processes that may regulate these traits. The results will improve our understanding of the molecular processes involved in muscle development and fat deposition of ruminants.

## Results

### Phenotypes and sequencing data

The phenotypic values of REA (cm^2^) and BFT (mm), animal identification, GEBVs (genomic estimated breeding values), the number of raw reads, and number and percentage of reads mapped against the *Bos taurus* UMD3.1 reference genome are shown in Tables 1 and 2. The heritability values for REA and BFT were 0.22 and 0.20, respectively [22]. There was no difference between the REA and BFT groups in regarding the intramuscular fat content, as well as the animals selected for REA were not significantly different for BFT and vice-versa (Additional file 1: Table S1). The correlation between REA and BFT in the sample of animals with contrasting GEBV (*n* = 22) tended to be low (*r* = −0.14).

**Table 1:** Phenotypic data, GEBV, the number of raw-reads, number of reads after cleaning, number and percentage of mapped reads for High and Low groups of ribeye area (REA)

Animal ID	REA (cm^2^)	BFT (mm)^a^	GEBV REA^b^	Raw reads^c^	Reads^d^	Mapped reads^e^	%^f^
HighREA^1^	72.00	15.00	4.71	9.75	7.66	6.68	87.19
HighREA^2^	79.75	4.00	3.47	17.10	15.17	11.42	75.30
HighREA^3^	66.75	4.00	3.26	25.92	21.33	12.83	60.15
HighREA^4^	66.20	3.50	3.22	21.97	16.22	10.13	62.47
HighREA^5^	73.25	5.00	3.22	11.81	6.91	5.49	79.52
HighREA^6^	73.25	6.00	2.92	25.60	15.97	12.77	79.96
LowREA^1^	51.25	9.50	−2.79	19.93	11.70	9.59	81.98
LowREA^2^	52.00	6.00	−2.92	12.47	7.15	5.38	75.30
LowREA^3^	42.50	9.00	−3.45	13.29	7.71	6.20	80.43
LowREA^4^	48.50	6.00	−3.54	17.83	10.78	8.13	75.40
LowREA^5^	50.75	8.00	−3.88	18.21	13.71	9.35	68.24
LowREA^6^	52.50	5.00	−3.95	11.31	6.61	5.26	79.59
Mean High	71.87	6.25	3.47	18.69	13.88	9.89	74.10
Mean Low	49.58	7.25	−3.42	15.51	9.61	7.32	76.84

^a^Backfat thickness

^b^genomic estimated breeding values for REA

^c^ millions of raw reads

^d^millions of reads after cleaning

^e^millions of mapped reads

^f^percentage of paired-end mapped reads

**Table 2:** Phenotypic data, GEBV, the number of raw-reads, number of reads after cleaning, number and percentage of mapped reads for High and Low groups of backfat thickness (BFT)

Animal ID	BFT (mm)	REA (cm^2^)^a^	GEBV BFT^b^	Raw reads^c^	Reads^d^	Mapped reads^e^	%^f^
HighBFT^1^	15.00	73.25	1.63	20.40	18.04	13.59	75.20
HighBFT^2^	14.00	56.75	1.63	12.36	8.49	5.57	65.59
HighBFT^3^	15.00	55.75	1.62	24.40	12.40	10.30	83.04
HighBFT^4^	11.00	56.25	1.48	16.22	13.95	10.58	75.90
HighBFT^5^	9.00	58.75	1.37	12.30	9.18	6.48	70.62
HighBFT^6^	15.00	62.50	1.19	25.60	15.97	12.77	79.96
LowBFT^1^	5.00	79.00	−0.88	16.98	15.09	11.51	76.30
LowBFT^2^	7.00	79.75	−0.91	24.89	21.32	16.07	75.40
LowBFT^3^	4.00	58.00	−1.02	17.13	14.74	11.25	76.30
LowBFT^4^	2.50	62.00	−1.03	8.79	5.11	4.08	79.92
LowBFT^5^	5.00	67.50	−1.06	17.10	15.17	11.42	75.30
LowBFT^6^	7.00	71.00	−1.17	11.75	7.02	5.86	83.55
Mean High	13.17	60.54	1.49	18.55	13.00	9.88	75.55
Mean Low	5.08	69.54	−1.01	16.11	13.07	10.03	75.83

^a^Ribeye area

^b^genomic estimated breeding values for BFT

^c^ millions of raw reads

^d^millions of reads after cleaning

^e^millions of mapped reads

^f^percentage of paired-end mapped reads

The choice of GEBV to select animals within extreme groups was made following Meuwissen et al. [25] and Sosnicki and Newman [26], who emphasized the importance of choosing genomic values as a vehicle to incorporate molecular information into selection programs. Also, the correlation between the GEBV and REA phenotypic values was high, *r* = 0.93. The same occurred for BFT, with GEBV and BFT correlation value of *r* = 0.90.

On average 76.34% of total paired reads aligned against the reference genome. After filtering, 18,468 and 18,411 genes were used for differential expression analysis, for REA and BFT, respectively.

### Differential expression analysis

**Fig. 1:**
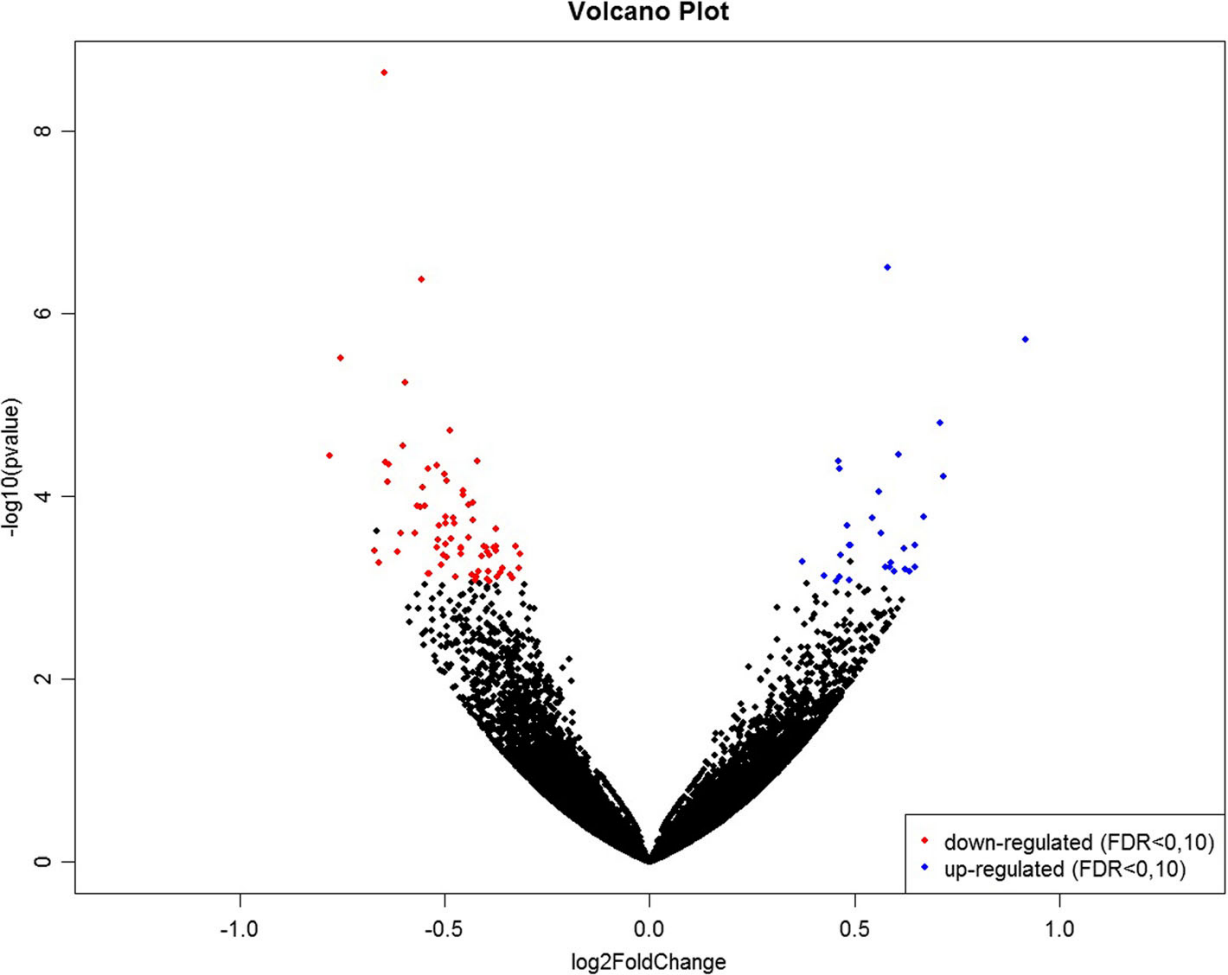
Volcano plot of log2FoldChange (x-axis) versus –log10 *p* value (FDR-corrected, y-axis) of high and low genomic breeding value groups for ribeye area in Nellore steers with FDR 10%

**Fig. 2:**
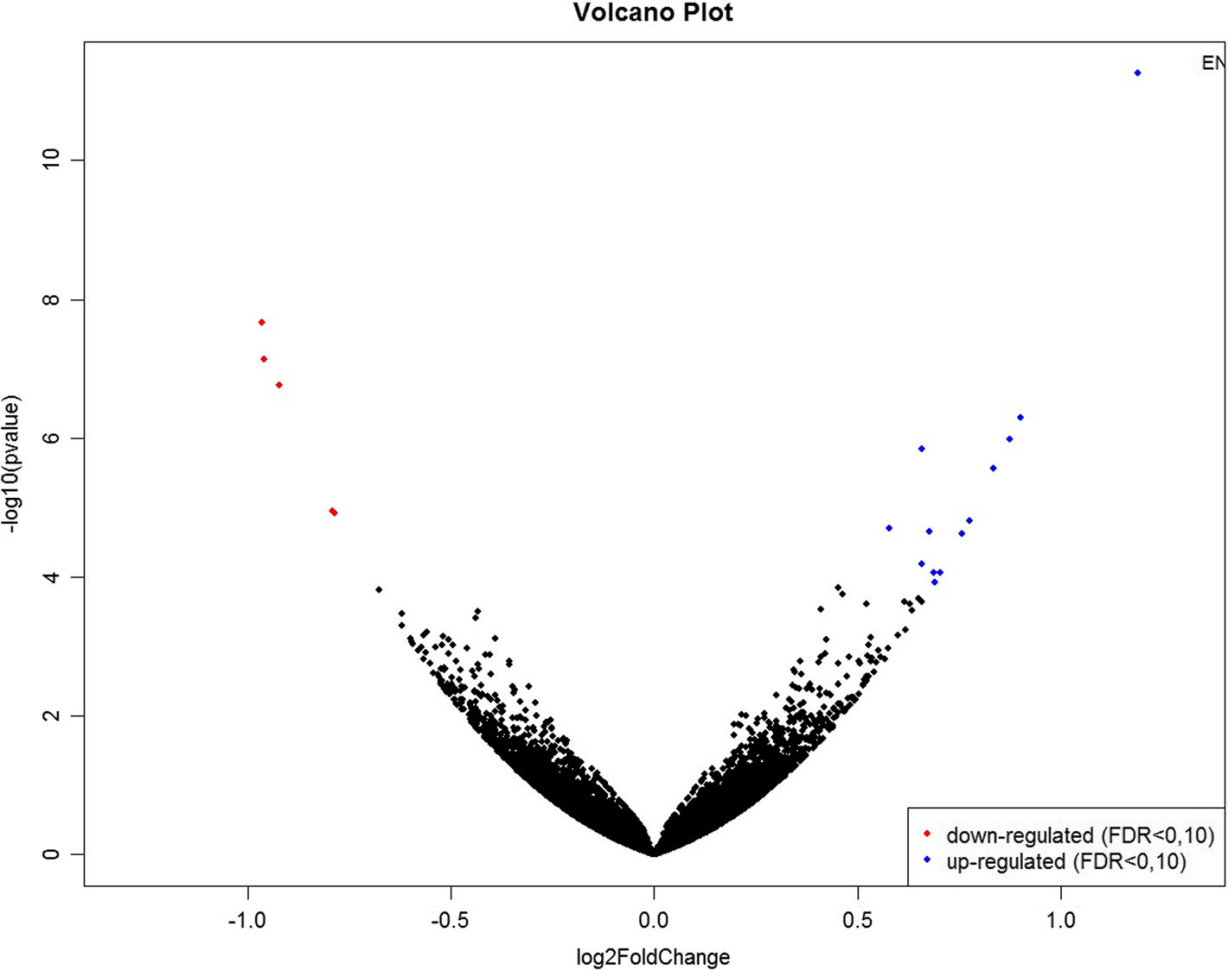
Volcano plot of log2FoldChange (x-axis) versus –log10 *p* value (FDR-corrected, y-axis) of high and low genomic breeding value groups for backfat thickness in Nellore steers with FDR 10%

Differential gene expression analysis between High and Low groups was conducted with DESeq2 software from R. DESeq2 uses statistical models based on a negative binomial distribution and is widely used to analyze RNA-Seq data since it allows more flexibility in assigning variations between samples [27]. One hundred and one differentially expressed (DE) genes were identified (false discovery rate, FDR 10%) between HighREA and LowREA groups, being 72 down-regulated and 29 up-regulated in the LowREA group. For BFT, 18 DE genes (FDR 10%) were identified, from which 13 were up-regulated and 5 were down-regulated in the LowBFT group. Figures 1 and 2 shows a Volcano plot of log2 foldChange (x-axis) vesus -log10 *p* value (FDR-corrected, y-axis) for REA and BFT, respectively. The gene annotation, log2foldChange, adjusted *p* value and *p* value of down- and up-regulated genes of REA and BFT can be found in Additional files 2: Table S2 and Additional file 3: Table S3, consecutively.

### Functional enrichment analysis

The functional enrichment analysis performed by DAVID (Database for Annotation, Visualization and Integrated Discovery) software identified two KEGG (Kyoto Encyclopedia of Genes and Genomes) pathways (*p* value <0.1) for REA: MAPK (Mitogen-Activated Protein Kinase) signaling pathway (bta04010) and endocytosis pathway (bta04144). The DE genes enriched for MAPK pathway were: *MAX* and *PPM1B* down-regulated*,* and *ARRB2, PTPRR* and *STMN1* up-regulated in the LowREA group. For the endocytosis pathway, the enriched genes were: *NEDD4* and *NEDD4L* down-regulated, *CHMP4A* and *ARRB2* up-regulated in the LowREA group. In the enrichment analysis performed by BINGO (Biological Networks Gene Ontology) software, three significant biological processes (FDR 5%) were identified for REA: response to endoplasmic reticulum stress (GO: 0034976), cellular protein modification process (GO: 0006464) and macromolecule modification (GO: 0043412). These BP can be seen in Table 3. Redundant terms were not found by REVIGO (Reduce + Visualize Gene Ontology).

**Table 3:** Significant biological processes (FDR 5%) identified by BINGO comparing high and low genomic breeding value groups for ribeye area

GO ID^a^	Description	p-adj.^b^	Log10 p-adj.^c^	Genes
GO:0034976	Response to endoplasmic reticulum stress	2.93 × 10^−2^	−1.5331	*PTPRR, NEDD4L, NEDD4, AMFR, UBE4A, NEK9, PPM1B, HOPX, EYA2, FES, TIE1, PCMTD1*
GO:0006464	Cellular protein modification process	2.93 × 10^−2^	−1.5331	*AMFR, CCDC47, COL4A3BP*
GO:0043412	Macromolecule modification	3.21 × 10^−2^	−1.4935	*PTPRR, NEDD4L, NEDD4, AMFR, UBE4A, NEK9, PPM1B, HOPX, EYA2, FES, TIE1, PCMTD1*

^a^Gene Ontology (GO) identification

^b^p value adjusted for a false discovery rate (FDR) of 5% [107]

^c^Log 10 of adjusted *p* value

For BFT, the functional enrichment analysis performed by DAVID identified five biological processes (*p* value <0.1) (Additional file 4: Table S4). The DE genes identified in these BP were *IDO1* and *ACHE*, down and up-regulated in the LowBFT group, respectively. The second enrichment analysis, performed by BINGO software, identified 18 significant biological processes (FDR 5%), grouped into eight clusters of semantically similar Gene Ontology (GO) terms by REVIGO (Table 4). The DE genes identified in the BP were *ACHE* and *SRD5A1* down-regulated, *RSAD2* and *RSPO3* up-regulated in the LowBFT group.

**Table 4:** Significant biological processes (FDR 5%) identified by BINGO comparing high and low genomic breeding value groups for backfat thickness

GO ID^a^	Description	p-adj.^b^	Log10 p-adj.^c^	Genes
**GO:0006702**	**androgen biosynthetic process**	**2.69 × 10** ^**−2**^	**−1.5702**	***SRD5A1***
GO:0008209	androgen metabolic process	4.48 × 10^−2^	−1.3487	*SRD5A1*
GO:0042446	hormone biosynthetic process	4.93 × 10^−2^	−1.3072	*SRD5A1*
**GO:0045213**	**neurotransmitter receptor metabolic process**	**3.23 × 10** ^**−2**^	**−1.4908**	***ACHE***
GO:0031623	receptor internalization	4.48 × 10^−2^	−1.3487	*ACHE*
GO:0032800	receptor biosynthetic process	4.48 × 10^−2^	−1.3487	*ACHE*
GO:0001919	regulation of receptor recycling	4.71 × 10^−2^	−1.3270	*ACHE*
**GO:0060713**	**labyrinthine layer morphogenesis**	**4.74 × 10** ^**−2**^	**−1.3242**	***RSPO3***
GO:0060669	embryonic placenta morphogenesis	4.71 × 10^−2^	−1.3270	*RSPO3*
GO:0060670	branching involved in labyrinthine layer morphogenesis	4.71 × 10^−2^	−1.3270	*RSPO3*
**GO:0042402**	**cellular biogenic amine catabolic process**	**4.74 × 10** ^**−2**^	**−1.3242**	***ACHE***
**GO:0060070**	**canonical Wnt signaling pathway**	**4.74 × 10** ^**−2**^	**−1.3242**	***RSPO3***
**GO:0008291**	**acetylcholine metabolic process**	**3.23 × 10** ^**−2**^	**−1.4908**	***ACHE***
GO:0006581	acetylcholine catabolic process	2.69 × 10^−2^	−1.5702	*ACHE*
GO:0042135	neurotransmitter catabolic process	4.48 × 10^−2^	−1.3487	*ACHE*
GO:0042133	neurotransmitter metabolic process	4.74 × 10^−2^	−1.3242	*ACHE*
**GO:0051607**	**defense response to virus**	**4.74 × 10** ^**−2**^	**−1.3242**	***RSAD2***
**GO:0045212**	**neurotransmitter receptor biosynthetic process**	**2.69 × 10** ^**−2**^	**−1.5702**	***ACHE***

^a^Gene Ontology (GO) identification

^b^p value adjusted for a false discovery rate (FDR) of 5% [107]

^c^Log 10 of adjusted *p* value

Terms highlighted in bold are the most representative of the similarity clusters by REVIGO analysis

## Discussion

Understanding how growth and development work may provide elements for increasing the profit and quality of meat production [28, 29]. Growth in livestock occurs mainly as a function of deposition of muscle and adipose tissue in the animal’s body [23]. As mentioned before, the ribeye area is a direct indicator of animal’s muscular development and has been used to predict the amount of lean meat in the carcass. In the other hand, the backfat thickness is used as an indirect indicator of meat in the carcass, and is very important to predict animal’s total body fat [3–5, 30, 31].

There are several biological processes involved in animal and muscle growth, such as the coordinated expression of many transcription factors (myogenic regulatory factors), genes and metabolic pathways, from the embryonic and fetal development until animals approach maturity. Most of the change in muscle weight during embryonic and fetal development is due to hyperplasia, the increase in number of muscle fibers. The postnatal stage of muscle growth (hypertrophy) consists in the increase in size of existing fibers. Both processes can be regulated by genetic factors, growth factors (insulin-like growth factors), hormones, and even environmental factors (mainly nutrition) acting as a positive or negative regulator of animal’s growth [29, 32, 33].

Furthermore, the processes of protein synthesis and degradation, also called protein turnover, affect muscle growth rates and can consequently alter carcass traits in beef cattle [23, 29, 34, 35]. During muscle hypertrophy, there is a balance between protein synthesis and degradation that may result in protein deposition, and therefore muscle growth [32]. Altogether, these processes will lead to differences in muscle and fat deposition, and hence animals with different proportions of REA and BFT.

### Ribeye area

The enrichment analysis performed by DAVID identified two pathways (KEGG). The first one was MAPK pathway, which is responsible for transduction of extracellular signals to their intracellular targets in various cell types, including skeletal muscle cells. This pathway acts in the control of fundamental cellular processes, such as proliferation, growth, migration, differentiation, apoptosis, and more specifically to muscle cells, hypertrophy [36–38]. According to Noordman, Jansen and Hendriks [39], the MAPK pathway is the main mechanism used by growth factors in processes such as cell proliferation and differentiation.

When activated, MAPKs phosphorylate several intracellular targets, which include numerous transcription factors, resulting in the reprogramming of gene expression and cell division [40]. The activity is regulated by autophosphorylation or by phosphorylation of other kinases. On the other hand, the inactivation occurs by the process of dephosphorylation, which can be initiated by protein tyrosine phosphatases (PTPs) and metal-dependent protein phosphatases (PPMs) [39, 41, 42].

The *PTPRR* gene (protein tyrosine phosphatase, receptor type R), up-regulated in the LowREA group, can act by regulating the dephosphorylation of MAPKs and inhibiting cellular processes of proliferation and differentiation [43, 44]. Li et al. [42], working with *PTPRR* expression in mice hippocampus, verified that a greater expression of this gene led to an increase in MAPK dephosphorylation and consequently neuronal apoptosis and a decrease in cellular proliferation, showing that this gene may be acting on inhibition of the MAPK pathway in the LowREA group.

The *PPM1B* (protein tyrosine phosphatase, Mg^2+^/Mn^2+^ dependent 1B) gene, down-regulated in LowREA group, encodes a protein of the PPM family and acts in MAPK pathway dephosphorylation. Wei and Liang [41] identified a negative correlation between *PPM1B* and muscle atrophy, that is, *PPM1B* expression gradually decreased when muscle atrophy increased.

The second pathway found in the present study was endocytosis, which is fundamental for eukaryotic cells and is highly conserved between species and cell types. Endocytosis acts on the regulation of several processes, like cell adhesion and migration, extracellular signal transduction, cell growth and differentiation [45]. Junior et al. [24] also found genes involved in cell cycle regulation and transportation of cellular substances associated with REA in Nellore cattle.

Four genes were enriched in the endocytosis pathway: *CHMP4A, ARRB2, NEDD4* and *NEDD4L.* Within them, *NEDD4* (neural precursor cell expressed, developmentally down-regulated 4, E3 ubiquitin protein ligase) and *NEDD4L* (neural precursor cell expressed, developmentally down-regulated 4-like, E3 ubiquitin protein ligase), also known by *NEDD4–2*, encode ubiquitin proteins ligases belonging to the Nedd4 family. Among their functions, they may aid protein internalization in the cells [46–48].

In addition to the protein internalization function, *NEDD4* is required for cell surface expression of the IGF-1R (insulin-like growth factor, type 1 receptor) and insulin receptor, and is a positive regulator of IGF-1 (insulin-like growth factor, type 1) and insulin signaling [46, 47]. In mammals, the Insulin-like growth factors (IGF) axis is the largest fetal and postnatal growth regulator and is strongly related to muscle differentiation [32, 49, 50]. Studies with knockout mice for the *NEDD4* gene showed that loss of *NEDD4* reduced IGF-1 and insulin signaling, delayed embryonic development, and reduced growth and body weight [46]. Junior et al. [24] in a GWAS study associated with REA, BFT and hot carcass weight found this gene enriched for the GO terms “cellular protein metabolic process” and “protein metabolic process” related to protein turnover and, consequently animal growth and development. In the present study, *NEDD4* and *NEDD4L* were down-regulated in LowREA group, emphasizing their importance in regulating muscle growth.

*ARRB2* (arrestin β-2), identified in both pathways – endocytosis and MAPK –, was up-regulated in the LowREA group. β-arrestins are multifunctional signaling molecules ubiquitously expressed that act as endocytosis regulators in different types of cell surface receptors [51, 52]. According to Luttrell and Lefkowitz [51], β-arrestins can serve as scaffold proteins for MAPK pathway proteins. Additionally, Yan et al. [53] show the involvement of β-arrestin 2 in the activation of MAPK pathway.

Analysis with BINGO software ascertained three biological processes: response to endoplasmic reticulum stress (GO: 0034976), cellular protein modification process (GO: 0006464) and macromolecule modification (GO: 0043412) (Table 3). Among the genes identified in these BP, *AMFR* (autocrine motility factor), a down-regulated gene in LowREA group, appears in all of them. *AMFR* – also known as *gp78* – encodes a *RING* (Really Interesting New Gene) class E3 ubiquitin protein ligase that is involved in the mechanism of protein quality control, eliminating misfolded proteins from the endoplasmic reticulum of eukaryotic cells [54–56].

The endoplasmic reticulum (ER) is a ubiquitous multifunctional organelle, which ensures the correct protein formation, and plays a key role in lipids and sterols synthesis, and in intracellular calcium maintenance [57]. ER stress can occur due to perturbations in its homeostasis, such as chemical damage, gene mutations, nutrient insufficiency, cell differentiation, oxidative stress, and fluctuation in calcium concentrations, leading to changes in protein structure, resulting in the accumulation of misfolded proteins in the ER lumen [58, 59]. ER stress can alter gene expression and cause post-transcriptional modifications, change cell physiology, and even induce cell apoptosis [57, 60, 61].

According to Nakanishi, Sudo and Morishima [61] and Nakanishi, Dohmae and Morishima [62] the ER response to stress related to the induction of apoptosis may be favorable for myogenesis. Nakanishi, Dohmae, and Morishima [62] working with mouse myoblast cells (C2C12) demonstrated that apoptosis induced by ER stress controls the differentiation of myoblasts, so only cells resistant to apoptosis undergo terminal differentiation to muscle tissue formation, improving the myoblasts quality.

The other two biological processes identified in the present study - cellular protein and macromolecule modification processes - are intrinsically related, since proteins can be classified as macromolecules. According to Cantin and Yates III [63], most proteins need to undergo modifications to carry out their activities or become biologically active, and these changes are called post-translational modifications (PTM).

PTMs are chemical changes that modify the protein structure reversibly or irreversibly, through proteolytic cleavage or covalent modifications in specific amino acid residues [64–66]. According to Blom et al. [65] and Zou and Blank [67], phosphorylation is the primary protein modifier and is considered a key event in several transductional signaling cascades, such as the MAPK pathway. As discussed previously, *PTPRR* and *PPM1B*, identified in the MAPK pathway, were also identified in the macromolecular modification process. These two genes encode protein phosphatases that can act by dephosphorylating and thus decreasing the activity of MAPK proteins [39, 41, 42].

Another gene found in the macromolecular modification process that encode a protein phosphatase is *EYA2* (EYA transcriptional coactivator and phosphatase 2). Unlike *PTPRR* and *PPM1B*, this gene acts as transcription factor inducing myogenic regulatory factors, such *MEF3* and *MYOG,* as well has important roles in differentiated muscle cells [68, 69]. In our finds, *EYA2* was down-regulated in LowREA group, showing its importance as a positive regulator of muscle growth.

Another common PTM is ubiquitination, which acts in directing short-life proteins to the proteasome degradation pathway [34, 54–56]. This ubiquitin-dependent proteolysis ensures protein turnover that is essential to cell survival [70]. In addition, the ubiquitination process also functions on cellular processes like signal transduction, enzymatic activation, endocytosis, molecular trafficking, chromatin rearrangement and DNA repair [71]. Among the genes identified in the cellular protein modification and macromolecular modification processes that encode ubiquitin proteins, *NEDD4* and *NEDD4L* already had their functions in muscle discussed here. The *UBE4A* (ubiquitin conjugation factor E4 A), down-regulated in the LowREA group, is an important and ubiquitously expressed gene for the ubiquitination process, that may also participate in growth and differentiation processes [72]. Gomes et al. [23] have already reported that genes related to protein turnover were associated with growth and carcass traits in Nellore cattle. Latterly, Junior et al. [24] also found GO terms related to protein turnover in an association study with REA, BFT and hot carcass weight in Nellore. These findings show us the important role of ubiquitination and ubiquitin proteins for muscle growth, and consequently to improve REA in the animals.

### Backfat thickness

The enrichment analysis performed by BINGO software identified 18 biological processes (FDR 5%), which were grouped into eight clusters of semantically similar GO terms by REVIGO (Table 4).

Acetylcholinesterase gene (*ACHE*), present in four similarity clusters and up-regulated in the LowBFT group, is an essential component of the neuromuscular junction (NMJ). This enzyme is highly conserved in mammals and appears in multiple molecular forms, which originate from the alternative splicing of *ACHE* gene [73, 74]. *ACHE* expression in muscle is regulated by transcriptional and post-transcriptional events, with an increased expression in the early stages of myogenic differentiation, and reaching a plateau when the myotubes are mature [74, 75].

Mouisel et al. [76] reported a loss of muscular weight in the hind limb muscles of *ACHE* knockout mice. Soysal et al. [77] concluded that acetylcholinesterase inhibitors could cause weight loss and change muscle mass index in elderly people. Despite not showing a direct relation with BFT, it is clear that this gene has a role during animal’s muscle growth.

The *SRD5A1* (steroid-5-alpha-reductase, alpha polypeptide 1), also known as *5α-reductase type 1*, was identified in the androgen biosynthetic process (GO:0006702). Androgens, such as testosterone, play a critical role in muscle, increasing protein synthesis and energy metabolism, and promoting growth and muscle strength increase [78, 79]. Ferrando et al. [79] hypothesized that testosterone might stimulate IGF-1 release in muscle tissue. Although skeletal muscle can synthesize and metabolize testosterone, this action on target organs often requires its metabolic conversion to one or more active products [80], such as DHT (dihydrotestosterone) metabolized by the 5α-reductase enzyme. DHT is one of the most potent natural androgens because of its high affinity for androgen receptors; it has several physiological effects on skeletal muscle, like activation of signaling pathways and anabolic action in protein synthesis, as well as the maintenance of muscle homeostasis [81–84].

Several studies found an association of *SRD5A1* and its corresponding protein (5α-reductase) with muscle weight and strength [83–86]. Sato et al. [86] identified a positive correlation of 5α-reductase protein with the cross-sectional area of *quadriceps femoralis* muscle in humans. Although not DE for REA, *SRD5A1* was up-regulated in the LowBFT group, that is, the group that presented a higher proportion of muscle mass represented by higher values of REA (Table 2). In contrast, Sun et al. [87] studying putative target genes for miRn25 and n26, highly expressed miRNAs in bovine backfat thickness, identified *SRD5A1* related to lipid synthesis in adult animals.

The R-spondin 3 gene (*RSPO3*), down-regulated in the LowBFT group, encodes a member of a protein family widely recognized as an agonist of the canonical Wnt signaling pathway (or Wnt/β-catenin pathway) [88–91], one of the biological processes found here enriched for this gene (GO:0060070). This pathway plays an essential role during embryonic muscle development and in skeletal muscle homeostasis during adulthood [89, 90, 92]. This pathway also is an important regulator of adipocyte differentiation [93, 94].

Han et al. [88] studied the role of RSPOs (r-spondins) during myogenic differentiation using primary satellite cells and C2C12 cells from mouse myoblast. The authors observed that silencing *RSPO2* and *RSPO3* significantly affected the *Myf5* expression, the rate of myogenic differentiation and the myotubes formation in mice muscle cells. The authors also found that RSPOs can act via canonical Wnt signaling pathway in the positive regulation of myogenesis in skeletal muscle.

Li et al. [93] studying adipose-derived mesenchymal stem cells in porcine found that the activation of Wnt signaling pathway suppressed mRNA and protein expression of the adipocyte-specific genes *C/EBPα* (CCAAT/enhancer-binding protein-α) and *PPARγ* (peroxisome proliferator-activated receptor-γ), inhibiting adipogenesis in these cells. Chen et al. [94] also found that Wnt signaling pathway may inhibit adipogenic differentiation in porcine intramuscular preadipocytes. So, even identifying this gene down-regulated in the LowBFT group, it is likely that it was acting as a negative regulator of BFT in the animals.

*RSAD2* gene (radical domain of S-adenosyl methionine containing 2), found in the defense response to virus process (GO:0051607), is a type I interferon response gene, which has been used in the clinical prediction of some diseases, also related to skeletal muscle myopathies caused by inflammatory cytokines [95–97]. Wei et al. [98] working with growing pigs fed a linseed-enriched diet, found *RSAD2* up-regulated in the *Longissimus dorsi* muscle of treated animals. Dogan et al. [99], studying the structure and composition of mice fat, muscle, and liver, reported that *RSAD2* may act as a modulator of lipid droplet content and lipid biosynthesis in adipose tissue. These findings coincide with this work, in which the *RSAD2* gene was down-regulated in the LowBFT group.

## Conclusions

Our results emphasize the complexity of gene regulation in the *Longissimus dorsi* muscle of Nellore cattle associated with REA and BFT. We identified 101 DE genes in the extreme GEBV groups for REA. These genes were enriched for metabolic pathways and biological processes mostly involved in differentiation, the proliferation of muscle cells, protein turnover and hypertrophy, such as the MAPK pathway, cellular protein, and macromolecule modification processes. For BFT, we identified 18 DE genes involved in biological processes that may regulate positively or negatively adipogenesis, lipid biosynthesis and muscle growth. These results might help us to enlighten the molecular processes involved in muscle and fat deposition, which are economically important carcass traits for beef production.

## Methods

### Animals, samples, and phenotypes

Three hundred eighty-five (385) Nellore steers from Embrapa (Brazilian Agricultural Research Corporation) breeding herd, raised between 2009 and 2011, were included in this study. To breed this herd, 34 unrelated bulls were selected representing the main breeding lineages used in Brazil based on the information of the National Summary of Nellore produced by the Brazilian Association of Zebu Breeders (ABCZ) and the National Research Center for Beef Cattle.

The animals were raised in grazing systems, under the same conditions of handling and nutrition until 21 months of age when they were taken to feedlots. All animals were slaughtered at an average age of 25 months. The slaughter was carried out in a commercial slaughterhouse located in the city of Bariri (São Paulo), under the supervision of the Federal Inspection Service (SIF) and within the standards established by the Brazilian Ministry of Agriculture, Livestock and Food Supply (MAPA), for more details see [100]. At the time of slaughter, approximately 5 g of the *Longissimus dorsi* muscle was collected between the 12th and 13th ribs (right half carcass) and were stored in liquid nitrogen. Twenty-four hour after slaughtering, steaks corresponding to a cross section of the *Longissimus dorsi* muscle between the 12th and 13th ribs (left half carcass) were sampled with bone and transported to the laboratory of Embrapa Pecuária Sudeste (São Carlos, SP), where REA and BFT were measured. REA was measured with a grid and the BFT with a graduated ruler.

The genomic estimated breeding values (GEBV) were obtained by the GenSel program [101], which uses Bayesian methodology. The a priori values of genetic and residual variance were obtained from the Bayes C analysis, in which the a priori genetic and residual variance was equal to 1 [102]. Using the estimated a priori values, a new Bayes C analysis was performed to obtain GEBVs for each animal. The SNP markers information was obtained as described by Cesar et al. [103] using BovineHD 770 k BeadChip (Infinium BeadChip, Illumina, San Diego, CA, USA). For BFT, 384 animals were used in the GEBV estimate. The animals were separated into two groups of six animals each (High and Low), based on the extreme values of GEBVs for each of the two traits. Of the 12 animals selected for each trait, two of them were in common for both traits (Additional file 5: Table S5). A Student’s t-test was performed to verify the difference in REA and BFT level between High and Low groups. The phenotypic values of intramuscular fat content were also included in this test to ascertain these animals were not significantly different for this trait. The phenotypic correlation between REA and BFT using the selected animals (*n* = 22) was estimated with the R software.

### RNA extraction, quality analysis, library preparation and sequencing

The total RNA was extracted from 100 mg of frozen *Longissimus dorsi* muscle collected at the slaughter using the TRIzol reagent (Life Technologies, Carlsbad, CA, USA), following the manufacturer’s instructions. At the end of the extraction process, RNA integrity was verified by the Bioanalyzer 2100 (Agilent, Santa Clara, CA, USA). The mean RIN (RNA integrity number) of all samples was 7.75. For library preparation, 2 μg of RNA from each sample was used, according to the protocol described in the TruSeq RNA Sample Preparation kit v2 guide (Illumina, San Diego, CA, USA). The libraries were quantified by quantitative PCR using the KAPA Library Quantification kit (KAPA Biosystems, Foster City, CA, USA) and the average library size was estimated using the Bioanalyzer 2100 (Agilent, Santa Clara, CA, USA). After quantification, the samples were diluted and pooled into three pools out of six samples each. Three lanes of a sequencing flowcell were clustered, using the TruSeq PE Cluster kit v3-cBot-HS (Illumina, San Diego, CA, USA). They were sequenced on the HiSeq2500 ultra-high throughput sequencing system (Illumina, San Diego, CA, USA) using the TruSeq SBS kit v3-HS (200 cycles), according to the manufacturer’s instructions. All sequencing analyses were performed at the Genomics Center at the College of Agriculture “Luiz de Queiroz” of the University of São Paulo.

### Quality control and read alignment

The adapter sequences and low complexity reads were removed in an initial data-filtering step using SeqClean software (https://sourceforge.net/projects/seqclean/files/). The FastQC software was used to analyze the quality of raw reads (http://www.bioinformatics.babraham.ac.uk/projects/fastqc/). The Tophat version 2.1.0 software [104] was used to map the reads against to the UMD3.1 *Bos taurus* reference genome (http://www.ensembl.org/Bos_taurus/Info/Index/). Read counts (mRNA abundance) for all mapped genes were calculated using the HTSeq version 0.6.1 software (http://www-huber.embl.de/HTSeq) [105]. Only read sequences that uniquely mapped to known chromosomes were used in this study.

### Identification and annotation of differentially expressed genes

Differentially expressed genes were identified using the DESeq2 software of R [106]. Before the statistical analysis was performed, the read count data was filtered based on previous studies [16, 17], as follows: i) genes with zero counts were removed (not expressed); ii) genes with less than one read per sample on average were removed (very low expression level); iii) genes that did not appear in at least three samples were removed (rarely expressed). After filtering, a total of 18,468 genes for REA and 18,411 for BFT were analyzed for differential expression employing the “nbinomWaldTest” function of the DESeq2 that assumes the level of gene expression as a negative binomial distribution. Exploratory plots were made to check the dispersion estimates (Additional file 6: Figures S1 and S2, and Additional file 7: Figures S3 and S4). The Benjamini and Hochberg [107] methodology was used to control the false discovery rate (FDR) at 10%. The DE genes were annotated by the online tool BioMart (http://www.ensembl.org/biomart) from Ensembl. Genes that lacked annotation information were annotated using the NCBI (National Center for Biotechnology Information - http://www.ncbi.nlm.nih.gov/) and Panther (http: /www.pantherdb.org) databases.

### Functional enrichment analysis

The functional enrichment analysis of DE genes (FDR 10%) for KEGG (Kyoto Encyclopedia of Genes and Genomes) pathways was carried out with the online tool DAVID (Database for Annotation, Visualization and Integrated Discovery) version 6.7 [108].

Also, another enrichment analysis was performed to identify biological processes related to the DE genes, using BINGO (Biological Networks Gene Ontology) version 3.0.3 [109], a Cytoscape [110] version 3.4.0 app. BINGO is a free-use tool that determines GO terms that are over-represented in a set of genes using the “Hypergeometric test” as a statistical test. BPs that presents FDR 5% [107] were considered significant. Lastly, REVIGO (Reduce + Visualize Gene Ontology), an algorithm that summarizes long lists of GO terms was used to remove redundant GO terms. REVIGO performs a simple clustering procedure finding a representative subset of GO terms that is based on semantic similarity measures [111].

## Additional files

Additional file 1: Table S1. Test of means (t-test) of intramuscular fat (IMF), backfat thickness (BFT) and ribeye area (REA) between groups with High and Low REA and BFT in the *Longissimus dorsi* muscle of Nellore steers. (XLS 33 kb)

Additional file 2: Table S2. Differentially expressed genes obtained between high and low genomic breeding value groups for ribeye area in Nellore steers with FDR 10%. The table contains the Ensembl gene identification, gene symbol, mean normalized counts, Log2 fold Change from low to high group, *p* value and *p* value adjusted for a false discovery rate of 10%. (XLS 45 kb)

Additional file 3: Table S3. Differentially expressed genes obtained between high and low genomic breeding value groups for backfat thickness in Nellore steers with FDR 10%. The table contains the Ensembl gene identification, gene symbol, mean normalized counts, Log2 fold Change from low to high group, *p* value and *p* value adjusted for a false discovery rate of 10%. (XLS 31 kb)

Additional file 4: Table S4. Functional enrichment analysis performed by DAVID (*p* value <0.1) comparing high and low genomic breeding value groups for backfat thickness. The table contains Gene Ontology category, identification and description, *p* value, *p* value adjusted for a false discovery rate of 10% and the DE genes for each category. (XLS 29 kb)

Additional file 5: Table S5. Correspondence between sample names and European Nucleotide Archive (ENA) identifier (accession PRJEB13188 and PRJEB19421). (XLS 29 kb)

Additional file 6: Figures S1 and S2. Plots of dispersion estimates of all ribeye area and backfat thickness genes. The black dots are the dispersion estimates of the empirical values of each gene; the red line represents the trend line; the blue dots represent the genes estimates regressed through the trend line used in the hypothesis test; and the blue circles above the “cloud” of points are genes which have high gene-wise dispersion estimates which are labelled as dispersion outliers, and will not be used in the hypothesis test. (PDF 110 kb)

Additional file 7: Figures S3 and S4. Histogram of *p*-values from RNA-Seq data of *Longissimus dorsi* muscle of Nellore steers by DESeq2 software, for ribeye area and backfat thickness. Y-axis represents the frequency of *p*-values and x-axis represents the residual of *p*-values. (PDF 108 kb)

## Abbreviations

BFT: Backfat thickness
BINGO: Biological Networks Gene Ontology
BP: Biological process
DAVID: Database for Annotation, Visualization and Integrated Discovery
DE: Differentially expressed
DHT: Dihydrotestosterone
ER: Endoplasmic reticulum
FDR: False discovery rate
GEBV: Genomic estimated breeding value
GO: Gene ontology
GWAS: Genome-wide association study
IGF: Insulin-like growth factor
KEGG: Kyoto Encyclopedia of Genes and Genomes
MAPK: Mitogen-Activated Protein Kinase
NMJ: Neuromuscular junction.
PPM: Metal-dependent protein phosphatase.
PTM: Post-translational modification.
PTP: Protein tyrosine phosphatase.
QTL: Quantitative trait loci.
REA: Ribeye area.
REVIGO: Reduce + Visualize Gene Ontology.
RSPOs: r-spondins.
SNP: Single nucleotide polymorphism.
