# Supplementary material for: Comparative muscle transcriptome associated with carcass traits of Nellore cattle
Source: BMC Genomics. 2017 Jul 3;18:506. doi: 10.1186/s12864-017-3897-x (PMC5496360; doi:10.1186/s12864-017-3897-x)
Supplement: Supplementary file 6 — Plots of dispersion estimates of all ribeye area and backfat thickness genes. The black dots are the dispersion estimates of the empirical values of each gene; the red line represents the trend line; the blue dots represent the genes estimates regressed through the trend line used in the hypothesis test; and the blue circles above the “cloud” of points are genes which have high gene-wise dispersion estimates which are labelled as dispersion outliers, and will not be used in the hypothesis test. (PDF 110 kb) [file 12864_2017_3897_MOESM6_ESM.pdf]

Figure S1 – Plot of dispersion estimates of all ribeye area genes.

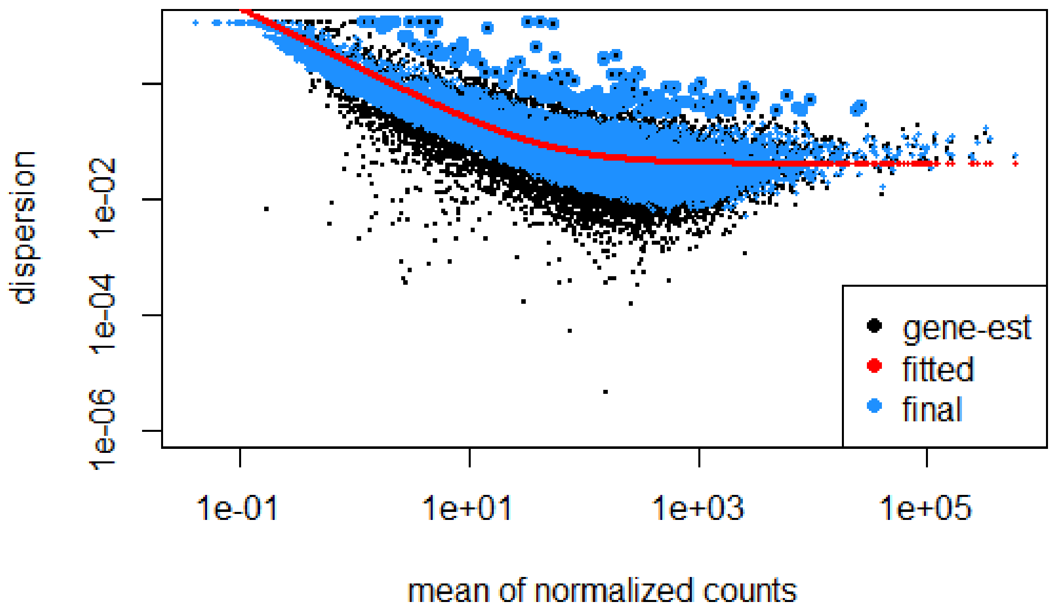

The black dots are the dispersion estimates of the empirical values of each gene; the red line represents the trend line; the blue dots represent the genes estimates regressed through the trend line used in the hypothesis test; and the blue circles above the "cloud" of points are genes which have high gene-wise dispersion estimates which are labelled as dispersion outliers, and will not be used in the hypothesis test.

Figure S2 – Plot of dispersion estimates of all backfat thickness genes.

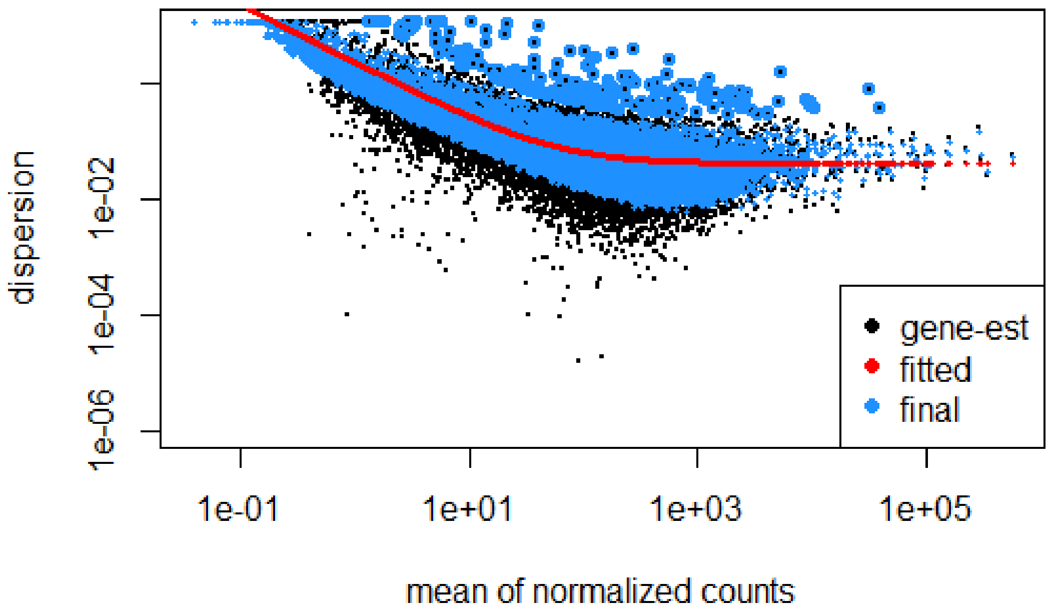

The black dots are the dispersion estimates of the empirical values of each gene; the red line represents the trend line; the blue dots represent the genes estimates regressed through the trend line used in the hypothesis test; and the blue circles above the "cloud" of points are genes which have high gene-wise dispersion estimates which are labelled as dispersion outliers, and will not be used in the hypothesis test.
