# Supplementary material for: Comparative muscle transcriptome associated with carcass traits of Nellore cattle
Source: BMC Genomics. 2017 Jul 3;18:506. doi: 10.1186/s12864-017-3897-x (PMC5496360; doi:10.1186/s12864-017-3897-x)

Figure S3 – Histogram of p-values from RNA-Seq data of *Longissimus dorsi* muscle of Nellore steers by DESeq2 software, for ribeye area.

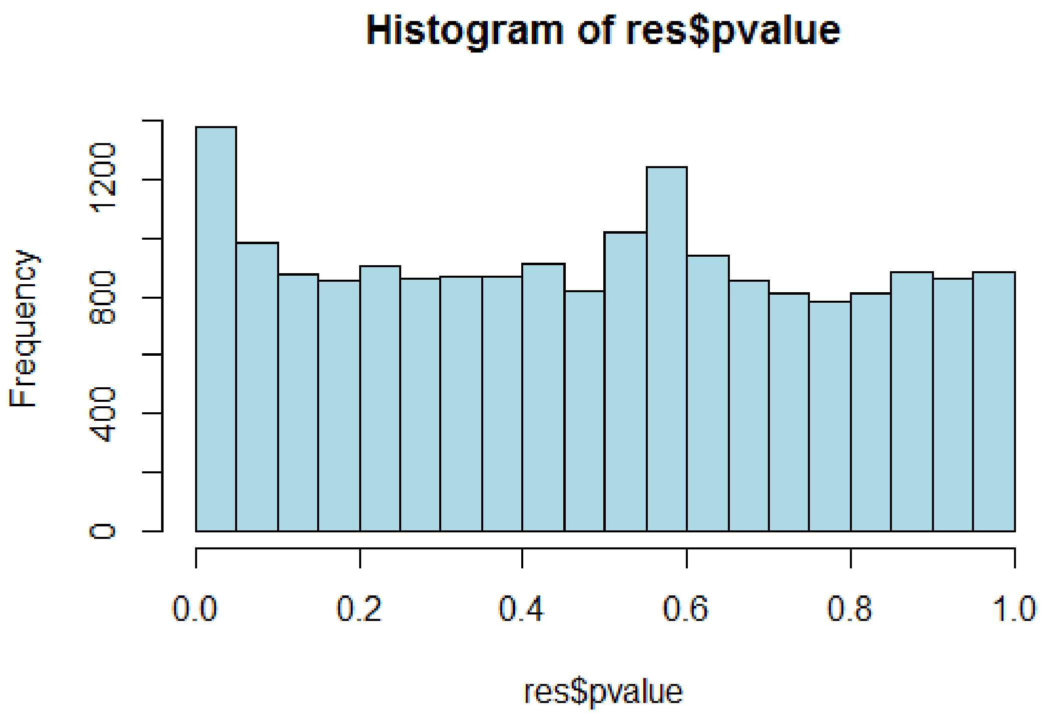

Figure S4 – Histogram of p-values from RNA-Seq data of *Longissimus dorsi* muscle of Nellore steers by DESeq2 software, for backfat thickness.

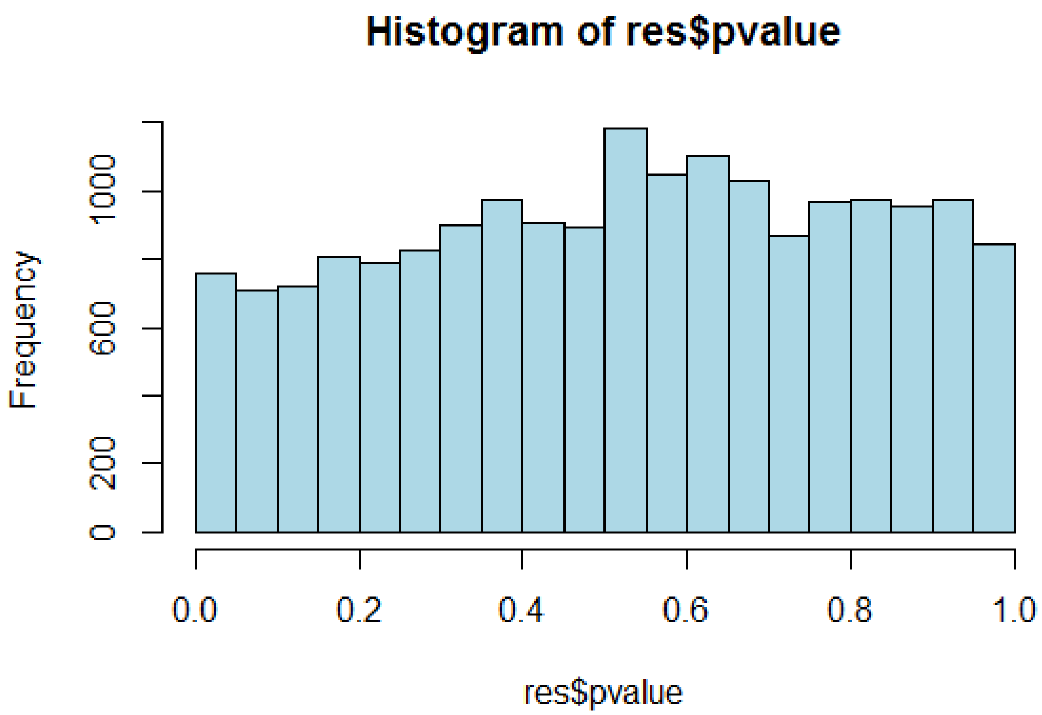

Supplement: Supplementary file 7 — Histogram of p-values from RNA-Seq data of Longissimus dorsi muscle of Nellore steers by DESeq2 software, for ribeye area and backfat thickness. Y-axis represents the frequency of p-values and x-axis represents the residual of p-values. (PDF 108 kb) [file 12864_2017_3897_MOESM7_ESM.pdf]
